# Etiology and Comorbidity Diagnoses Effect on Outcomes for Patients Undergoing Endoscopic Retrograde Cholangiopancreatography

**DOI:** 10.7759/cureus.10209

**Published:** 2020-09-02

**Authors:** Victoria Gordon, Anni Chowdhury, Sarah Keim

**Affiliations:** 1 Anatomy, Kansas City University of Medicine and Biosciences, Kansas City, USA; 2 General Surgery, The University of Kansas Medical Center, Kansas City, USA

**Keywords:** endoscopic retrograde cholangiopancreatography (ercp), nationwide inpatient sample (nis), outcomes

## Abstract

Introduction

Endoscopic retrograde cholangiopancreatography (ERCP) is now the first-line approach to treating choledocholithiasis. As a minimally invasive procedure, it is considered relatively safe but still entails a higher risk than other routine endoscopic procedures. This study aims to look at possible patient etiologies and comorbidities that may affect patient outcomes.

Methods

This study used the Nationwide Inpatient Sample (NIS) from the years 2012 - 2015 to collect anonymous patient data through the use of International Classification of Diseases, Ninth Revision (ICD-9) codes. Specific codes were used to determine the top five etiologies (or presenting diagnosis) for patients who had this surgery and to separate outpatients with specific comorbidity diagnoses. The IBM Statistical Package for Social Sciences (SPSS) (IBM SPSS Statistics, Armonk, NY) was then used to compare patients with these diagnoses or etiologies to those without to measure differences in patient outcomes, such as mortality, length of stay, and total charges.

Results

Patients who had an etiological diagnosis of acute kidney failure had worse outcomes than patients who were admitted for ERCP without that etiological diagnosis. There were also specific comorbidity diagnoses that were noted to have worse patient outcomes, including congestive heart failure, diabetes mellitus with complications, a coagulopathy disorder, anemia, or chronic liver disease. Additionally, patients who had both acute kidney disease and chronic liver disease had the worst outcomes.

Conclusions

This study highlights the need to understand all patient risk factors before having them undergo ERCP, especially in the setting of scheduled surgery. Working to control these factors before surgery can increase the possibility of avoiding negative outcomes like mortality, increased patient costs, and increased length of stay.

## Introduction

The foundations of endoscopic procedures were laid down over a century ago with the first open tube instrument developed in 1853 for examination of the bladder and urethra [1]. It was not until 1987 that the early method of making a single large incision via open cholecystectomy for the removal of gallbladder stones was largely replaced by the newer technique of inserting a scope through keyhole incisions, namely, laparoscopic cholecystectomy. The last few decades have seen impressive progress in methods of treating gallbladder diseases, including the extraction of gallstones in the biliary tree via endoscopic retrograde cholangiopancreatography (ERCP), which is now the first approach to treating choledocholithiasis. An estimated 350,000 to 500,000 ERCP procedures are performed annually in the United States for a myriad of gastrointestinal complaints in addition to choledocholithiasis, including malignant biliary obstruction, recurrent pancreatitis, obstructive jaundice, biliary and pancreatic strictures, and biliary leaks [2]. An assessment of the rate of stone clearance via the ERCP method from the Netherlands has shown a success rate of up to 85.2% in common bile duct stone clearance, making it a largely successful procedure [3]. However, among the remaining 14.8%, the causes of procedural failure have yet to be studied extensively. While therapeutic ERCP is minimally invasive and provides better outcomes to alternative surgical options, it still entails a higher risk of complications when compared to other routine endoscopic procedures, such as esophagogastroduodenoscopy and colonoscopy.

The aim of this study is to determine the specific factors that lead to adverse outcomes for patients undergoing ERCP for etiologies such as biliary stone disease without obstruction, biliary stone disease with obstruction, acute pancreatitis, acute kidney failure, and other gallbladder disorders. It is hypothesized that in-hospital outcomes, including the length of stay, total charges, and mortality rates of therapeutic endoscopic approaches, seem to be heavily influenced by existing comorbidities of patients undergoing them. The secondary comorbid conditions considered were congestive heart failure (CHF), diabetes mellitus (DM), coagulopathies, anemia, and chronic liver disease (CLD). DM was chosen based on previous studies that have linked perioperative hyperglycemia due to DM to adverse outcomes and surgical complications. Concomitant diabetes mellitus in patients undergoing ERCP is related to higher rates of postoperative infections (POI) as hyperglycemia increases the virulence of infectious microorganisms and causes apoptosis of polymorphonuclear neutrophils [4]. This leads to low neutrophil counts and diminished T-lymphocyte response, often causing bacteremia. POI leads to a higher need for inpatient care and higher total costs. Similarly, in patients with CLD, cirrhosis can contribute to higher surgical risk due to compromised liver function from the liver pathology itself and additional coagulopathies, anemia, and renal dysfunction that it can cause [4]. Procedure-related gastrointestinal bleeds are statistically more significant post-ERCP in compensated cirrhosis due to the development of coagulation disorders and low platelet counts, platelet dysfunction, and powerful collateral vasculature in the duodenal area as a result of portal hypertension [5]. The administration of an anesthetic for ERCP procedure in itself has adverse effects, such as a reduction in hepatic blood flow and oxygen intake. Resultant hypercarbia can decrease portal blood flow and reduce the metabolism of narcotics, sedatives, and antibiotics, decreasing drug clearance from the body. Perioperative anemia has historically shown a relationship with poor vascular and cardiac outcomes, lower survival rates post-infrainguinal surgeries, and increases the need for perioperative transfusions which come with its own risks, such as infectious complications, transfusion reactions, and higher rates of morbidity and mortality [6]. The longer postoperative recovery time and increasing length of hospital stay in anemic patients undergoing non-cardiac surgery add to total resource utilization and hospital costs [7]. In heart failure patients, clinical signs of volume overload, such as pulmonary edema and an S3 heart sound, have been linked to poorer outcomes. The physiological changes of heart failure include diastolic dysfunction with eccentric left ventricular remodeling, volume overload, and tachycardia. Patients undergoing non-cardiac interventions are believed to experience fluid shifts and high extravascular volume expansion, which could affect surgical outcomes, postoperative mortality, and hospital readmission rates [8-9]. 

The aim of this study was to determine what factors can lead to adverse in-hospital outcomes for patients undergoing ERCP and quantifies the effect of these comorbidities, along with the diagnosis of chronic kidney disease, on overall outcomes in ERCP patients.

## Materials and methods

Prospective data were collected from the Nationwide Inpatient Sample (NIS) from the years 2012 to 2015. From this dataset, all patients who underwent an endoscopic biliary stone removal, as described by an ICD-9 code, were selected. Relevant ICD-9 codes are included in Table 1. Patients under 18 or missing any age, cost, or death-related values were excluded from the sample.

**Table 1:** International Classification of Diseases, Ninth Revision (ICD-9) Codes

ICD-9 Code	Meaning
51.88	Endoscopic biliary stone removal
574.3	Calculus of bile duct with acute cholecystitis, without mention of obstruction
574.5	Calculus of bile duct without mention of cholecystitis, without mention of obstruction
574.6	Calculus of gallbladder and bile duct with acute cholecystitis, without mention of obstruction
574.7	Calculus of gallbladder and bile duct with other cholecystitis, without mention of obstruction
574.8	Calculus of gallbladder and bile duct with acute and chronic cholecystitis, without mention of obstruction
574.9	Calculus of gallbladder and bile duct without cholecystitis, without mention of obstruction
574.51	Calculus of bile duct without mention of cholecystitis, with obstruction
574.61	Calculus of gallbladder and bile duct with acute cholecystitis, with obstruction
574.71	Calculus of gallbladder and bile duct with other cholecystitis, with obstruction
574.91	Calculus of gallbladder and bile duct without cholecystitis, with obstruction
577.0	Acute pancreatitis
576.8	Other specified disorder of the biliary tract
576.1	Cholangitis
997.41	Retained cholelithiasis following cholecystectomy
584.9	Acute kidney failure, unspecified

The NIS database captures up to 30 diagnosis codes for each patient. These were used to determine the etiology and comorbidities that each patient was diagnosed with. When searching for the comorbidity diagnoses for each patient, all 30 diagnosis codes were used. Thirteen common comorbidities that affect surgical outcomes were looked at, and these included CHF, hypertension with and without complications, chronic pulmonary disease, DM with and without complications, coagulopathies, obesity, anemia, peripheral vascular disease, coronary artery disease, hyperlipidemia, and CLD. 

When searching for an etiology or admitting diagnosis, the first five diagnosis codes were used as they are more likely to be the initial admitting diagnosis. Any code that was used in more than 3% of patients was considered in the analysis. Some patients may fit into more than one etiology and/or comorbidity category.

The data were analyzed by the IBM Statistical Package for Social Sciences (SPSS) (IBM SPSS Statistics, Armonk, NY) in order to determine which etiologies and comorbidities had poor outcomes through chi-squared tests for evaluating mortality, independent t-tests for evaluating the length of stay, total charges, and analysis of variance (ANOVA) for comparing multiple variables, such as a comorbidity and etiology. Only if the etiology/admitting diagnosis or comorbidity led to statistically significantly poor mortality outcomes was other outcome data also assessed. P-values of less than .05 were considered to be statistically significant.

## Results

During the study’s timeframe, there were 53,244 cases of endoscopic biliary stone removal in the database remaining after those that met the exclusion criteria were removed. Controls were defined as the patients remaining in the population after those with the target etiology or comorbidity were identified and pulled for analysis.

Etiology or admitting diagnoses

There were 15 etiology codes that met the criteria for consideration. These codes were grouped into five categories. Category 1 was biliary stone disease without obstruction and included ICD-9 codes 574.3 and 574.5 - 574.9, and there were 24,903 patients included in this category. Category 2 was biliary stone disease with obstruction and included ICD-9 codes 574.51, 574.61, 574.71, and 574.91, and there were 11,766 patients included in this category. Category 3 was acute pancreatitis coded by ICD-9 code 577.0, and there were 13,068 patients included in this category. Category 4 was other disorders of the gallbladder and contained ICD-9 codes 576.1, 997.41, and 576.8, and there were 12,831 patients included in this category. Finally, Category 5 was acute kidney failure coded by the ICD-9 code 584.9, and there were 3,860 patients included in this category, leaving 49,385 patients without acute kidney failure as an etiological diagnosis.

Out of all of these codes, only patients in Category 5 had statistically significantly worse mortality outcomes. Those in Category 5 had statistically significantly higher rates of mortality (1.84% vs 0.86%, p < .0005) than those who were admitted without an etiological diagnosis of acute kidney failure. They also had longer length of stays (7.19 vs 5.14 days, p < .0005) and greater total charges ($69,822 vs $56,498, p < .0005) than those who were admitted without an etiological diagnosis of acute kidney failure.

Comorbidity diagnoses

The results are summarized in Table 2 for clarity. CHF was diagnosed in 4,037 patients, 1,514 patients had a diagnosis of DM with complications, 2,870 patients had a coagulopathy disorder, 8,116 patients were diagnosed with anemia, and 3,521 patients were diagnosed with CLD. All patients with the comorbidities chosen (those with a statistically significantly higher rate of mortality) also had longer lengths of stay and higher total charges compared to patients without these specific comorbidity diagnoses. 

**Table 2:** Comorbidity Outcomes

Comorbidity	Mortality	Length of Stay	Total Charges
Congestive Heart Failure	2.35% vs .81%, p < .0005	7.73 vs 5.09 days, p < .0005	$76,490 vs $55,903, p < .0005
Diabetes Mellitus with Complications	1.45% vs .92%, p = .029	7.49 vs 5.23 days, p < .0005	$76,234 vs $56,915, p < .0005
Coagulopathy	3.24% vs .80%, p < .0005	7.73 vs 5.15 days, p < .0005	$83,857 vs $55,960, p < .0005
Anemia	1.51% vs .84%, p < .0005	6.83 vs 5.01 days, p < .0005	$71,949 vs $54,859, p < .0005
Chronic Liver Disease	1.49% vs .83%, p < .0005	6.06 vs 5.24 days, p < .0005	$66,933 vs $56,793, p < .0005

Etiology and comorbidity diagnoses

Etiology and comorbidity diagnoses are summarized in Table 3. This table symbolizes patients who had the diagnosis of acute kidney failure upon admission and then also had another comorbidity diagnosis and whether having this negative etiology and a negative comorbidity further negatively impacted the patient. This table shows comparisons between patients without any of these diagnoses, patients with an admitting diagnosis of acute kidney failure and a comorbidity, patients without kidney failure, and none of the studied comorbidities, or patients with either acute kidney failure or a comorbidity diagnosis. Reported in the table are means and p-values. There were 755 patients who had CHF and were admitted with a diagnosis of acute kidney failure. Two hundred sixty-seven patients had a DM with complications and were admitted with a diagnosis of acute kidney failure. Five hundred eleven patients had a coagulopathy and were admitted with a diagnosis of acute kidney failure. One thousand one hundred and one patients had anemia and were admitted with a diagnosis of acute kidney failure. Three hundred and ten patients had CLD and were admitted with a diagnosis of acute kidney failure.

**Table 3:** Acute Kidney Failure Etiology/Admitting Diagnosis AND a Comorbidity 1) Mean of a patient with acute kidney failure and the comorbidity; 2) As compared to patients with neither acute kidney failure nor the comorbidity; 3) As compared to a patient with just kidney failure; 4) As compared to a patient solely experiencing the comorbidity

		Comorbidity			
	CHF	DM with complications	Coagulopathy	Anemia	CLD
Outcome					
Mortality	1) 2.52%	1) .37%	1) 3.32%	1) 1.54%	1) 3.55%
	2) .76%, p < .0005	2) .84%, p = .858	2) .74%, p = .004	2) .76%, p = .038	2) .83%, p < .0005
	3) 1.68%, p = .135	3) 1.95%, p = .048	3) 1.61%, p = .005	3) 2.0%, p = .622	3) 1.69%, p = .006
	4) 2.35%, p = .972	4) 1.68%, p = .180	4) 3.22%, p = .005	4) 1.48%, p = .997	4) 1.40%, p = .001
Length of Stay (days)	1) 8.19	1) 8.34	1) 8.57	1) 7.70	1) 8.44
	2) 4.96, p < .0005	2) 5.08, p < .0005	2) 5.02, p < .0005	2) 4.88, p < .0005	2) 5.09, p < .0005
	3) 6.95, p < .0005	3) 7.11, p = .004	3) 6.98, p < .0005	3) 6.99, p = .003	3) 7.08, p < .0005
	4) 7.62, p = .062	4) 7.31, p = .036	4) 7.55, p = .001	4) 6.69, p < .0005	4) 5.83, p < .0005
Total Charges ($)	1) 75,172	1) 79,815	1) 84,942	1) 73,318	1) 88,708
	2) 55,053, p < .0005	2) 56,007, p < .0005	2) 55,138, p < .0005	2) 53,976, p < .0005	2) 55,919, p < .0005
	3) 68,521, p = .092	3) 69,079, p = .078	3) 67,515, p < .0005	3) 68,427, p = .208	3) 68,173, p < .0005
	4) 76,793, p = .941	4) 75,467, p = .798	4) 83,621, p = .981	4) 71,734, p = .9	4) 64,830, p < .0005

While the length of stay and total charges are important metrics, mortality is most important in healthcare. Patients with chronic liver disease and an admitting diagnosis of acute kidney failure had the poorest prognosis across all categories and all combinations of patient diagnosis. The only other group that saw worse statistics when compared to the control were patients with coagulopathies who experienced statistically significantly higher mortality in all categories.

## Discussion

This study evaluated the intersection of etiology, comorbidities, and undergoing ERCP. It is well-documented that conditions like CHF, CLD, anemia, coagulopathies, and DM can negatively affect surgical outcomes in general [6-11], and in the case of CLD, previous negative outcomes have been directly linked to ERCP [5]. While most studies only look at mortality in reference to outcomes, this study also evaluated non-medical outcomes, such as total charges and patient length of stay (LOS). These outcomes are also important to patients as they cause undue stress and can cause adverse life circumstances, leading to further complications for patients. It is important to recognize that there are other negative outcomes important to patients besides mortality outcomes. Medical debt and the amount of time spent in the hospital are also concerns for patients.

Looking at each comorbidity individually, these were chosen for their known ability to negatively impact surgical outcomes. CLD, as stated, has been previously linked to negative ERCP outcomes, and this is likely due to its extensive part in producing clotting factors and processing toxins [4-5]. For similar reasons, patients with a coagulopathy disorder are likely to experience negative outcomes due to bleeding or clotting risks. Patients with anemia are more likely to need transfusions in the setting of surgery, and these can lead to a longer stay and elevated hospital costs which are negative patient outcomes discussed in this study [7]. CHF patients can experience cardiac dysfunction and volume expansion that can affect post-surgical healing [9]. DM can lead to a decreased immune response and increased virulence of infectious agents, meaning that any postoperative infection complication is more likely to lead to negative patient outcomes than for those without DM [4]. Due to factors related to each comorbidity, patients with each comorbidity are likely to experience the negative patient outcomes described in this study related to ERCP. Understanding why these comorbidities can lead to negative outcomes is important for understanding why it is important to continue to try to treat and control these diagnoses in patients prior to surgical intervention.

It was also noted, especially in Table 3, that there are additive negative effects when patients have more than one diagnosis. For example, having an etiological diagnosis of acute kidney failure and any one of the comorbidities usually led to worse outcomes in all categories than for patients without the comorbidity and without acute kidney failure. However, the most interesting finding was that patients diagnosed with CLD and acute kidney failure did worse than all of the other patients since having deficiencies in both of the two toxin removal systems in the body can cause negative outcomes during surgery. These patients could have clotting disorders due to liver dysfunction, as well as an inability for physicians to compensate by using medications that worked through a renal excretion mechanism [5]. Additionally, liver dysfunction can lead to renal dysfunction independently, making this an additive effect in the setting of CLD and acute kidney failure [4].

By building on the current set of literature regarding these comorbidities and etiology and studying them in a setting related to the ERCP procedure, physicians can use this information to make informed patient care decisions regarding performing a surgery that is usually elective and/or scheduled and can, therefore, be moved to accommodate treatment or control of any of these conditions. Physicians should consider alternative treatment options in the setting of these diagnoses to improve patient outcomes, long-term satisfaction, and quality of life.

This study was limited by several factors. Due to the fact that this is a database study, accuracy relies on proper and timely coding which was likely not always done. Additionally, there is no way to know what happened post-discharge or if a patient who was discharged and then readmitted was counted twice in the analysis. It is also possible that patients experienced some of these comorbidities and/or etiologies and it was just not known by their physician. Future studies should look more in-depth at each of these individual etiologies and comorbidities in order to determine how they affect the ERCP specifically. They should also look at other factors that may negatively affect patient outcomes in the setting of ERCP.

## Conclusions

This study highlights the importance of understanding all of the patient risk factors before undergoing ERCP, especially since this is normally an elective or pre-scheduled surgery. Being cognizant of these issues and working to control them beforehand can lead to drastic reductions in negative patient outcomes. It also should be recognized that mortality, while a terrible outcome, is not the only negative outcome that patients can experience. Extensive LOS or charges can derail a patient’s life as well, and anything physicians can do to prevent these negative outcomes, in addition to reducing mortality, is important. When performing ERCP, it is especially important to carefully consider surgery in patients with an admitting diagnosis of CKD and those with comorbidities, such as CHF, coagulopathies, DM with complications, CLD, and anemias, to maximize positive patient outcomes.
